# Analysis of the therapeutic effect of artificial leather embedding combined with fascial sleeve flap transplantation on chronic wounds of lower limbs with bone and plate exposure

**DOI:** 10.1186/s12893-022-01521-2

**Published:** 2022-02-26

**Authors:** Yong Li, Zhi-bo Zhang, Ji-song Liu, Zhu-min Wu, Xin-cheng Sun, Yu-tin Zhao, Xiang-zhou Zhang

**Affiliations:** Department of Burn and Plastic Surgery, Third Hospital of Bengbu, Bengbu, 233000 China

**Keywords:** Bone exposure, Chronic wound, Plate exposure, Artificial dermis, Flap

## Abstract

**Background:**

After severe trauma of lower limbs, bone, tendon or plate graft exposure is common. The traditional repair method is to use a variety of skin flap transplantation to cover the exposed part, but the wound often can not heal after operation, or the wound is cracked, ulcer, sinus, bone and steel plate are exposed again after wound healing. The reason for this result is that when the flap is covered, the space around the bone plate is not well closed, forming a dead cavity, blood and exudate accumulation, hematoma formation or infection, and finally the wound ruptures again. In addition, due to the swelling and contracture of the flap after operation, the suture tension between the flap and the receiving area becomes larger, the skin becomes thinner and broken, and then the wound is formed. In order to solve the above problems, we carried out the study of artificial true skin embedding combined with fascial sleeve flap transplantation in the treatment of chronic bone plate exposed wounds of lower limbs.

**Methods:**

In this paper, 11 cases of chronic wounds with bone exposure and skin necrosis after steel plate implantation were selected. First stage is the wound bed preparation including primary wound expansion, removal of necrotic tissue and incision of sinus wall, removal of deep necrotic bone and fibrotic scarred skin on the outer wall of steel plate to normal tissue on the outer edge of the wound, removal of precipitated peptone and purulent fur in the hole, periphery and bone space of the steel plate, and removal of tendon tissue with basal necrosis and disintegration of the wound. After vacuum sealing drainage (VSD) 1–2 weeks, the peritraumatic basal granulation tissue grew well and there was no necrotic tissue in the wound. In the second stage, the exposed bone was covered with artificial dermis, the steel plate hole or the periphery and the basal space were filled, and the exposed steel plate was completely embedded, and then the fascia sleeve flap was transplanted to cover the wound. The sural neurovascular flap was performed in nine cases and the lateral superior malleolar artery perforator flap in two case.

**Results:**

The flap survived well in all 11 cases. During the follow-up of 6 months to the removal of the plate, there was no case of rupture, exposure and sinus formation.

**Conclusions:**

Artificial dermal covering combined with fascial sleeve flap transplantation can effectively avoid wound dehiscence or sinus formation caused by foreign body retention, infection and flap contracture. It has good effect in repairing chronic wounds with bone plate exposure after severe trauma of lower limbs.

## Introduction

Most of the lower limb injuries are severe high-energy injuries, such as crush injury, electric burn, blast injury and so on, often accompanied by bone exposure, severe fracture and tendon injury. In some cases, bone, joint, tendon and steel plate are often exposed or form sinus due to ischemic necrosis caused by local skin and soft tissue swelling, or because of osteomyelitis, wound infection and other reasons [1–4] after orthopedic plate internal fixation. After a period of so-called observation and treatment, the wound failed to heal, and then turned to chronic wound. In clinical practice, we often use various flaps to repair this kind of wound, but it is still common that the exposure of the bones, joints and plates or the formation of sinus tract recur [1, 5]. In this study, after debridement and vacuum sealing draina (VSD) for wound bed preparation, artificial dermis (PELNAC, GUNZE LIMITED, JAPAN.) was used to cover exposed bone, embed exposed steel plate, filled the peripheral space of steel plate, and then fascia sleeve flap transplantation was performed, in consequence, the study produced good results.

## Data and methods

### General information

A total of 11 cases, 9 males and 2 female, aged from 18–55 years old. There were seven cases of traffic accident rolling, three cases of high-voltage electric injury and one case of falling from height. The wounds were located in the lower part of the tibia, bone exposure with sinus formation in three cases, including bone and steel plate exposure in six cases, steel plate exposure and chronic sinus in two cases. The time from injury to admission was 1–6 months.

### Cases selection

#### Inclusion criteria

Chronic wounds with skin and soft tissue defects of lower extremities and exposure of bone and steel plate, the blood vessels of lower limbs were unobstructed, there are enough donor sites of skin flap at the back of the leg, and all patients are in good condition and can tolerate the operation and anesthesia.

#### Excluded cases

Patients with severe systemic diseases and intolerant to surgery and anesthesia, severe vascular injury of lower extremities and not enough donor area, obvious worries about surgical methods, severe wound infection, osteomyelitis.

### Method CTA (Computed Tomography Angiography) or CDFI (color doppler flow imaging)

Method CTA (Computed Tomography Angiography) or CDFI (color doppler flow imaging) were used to check the patency of lower extremity artery and perforator before operation. Then the selected cases were operated as follows.

#### Wound bed preparation in first stage

**Fig. 1:**
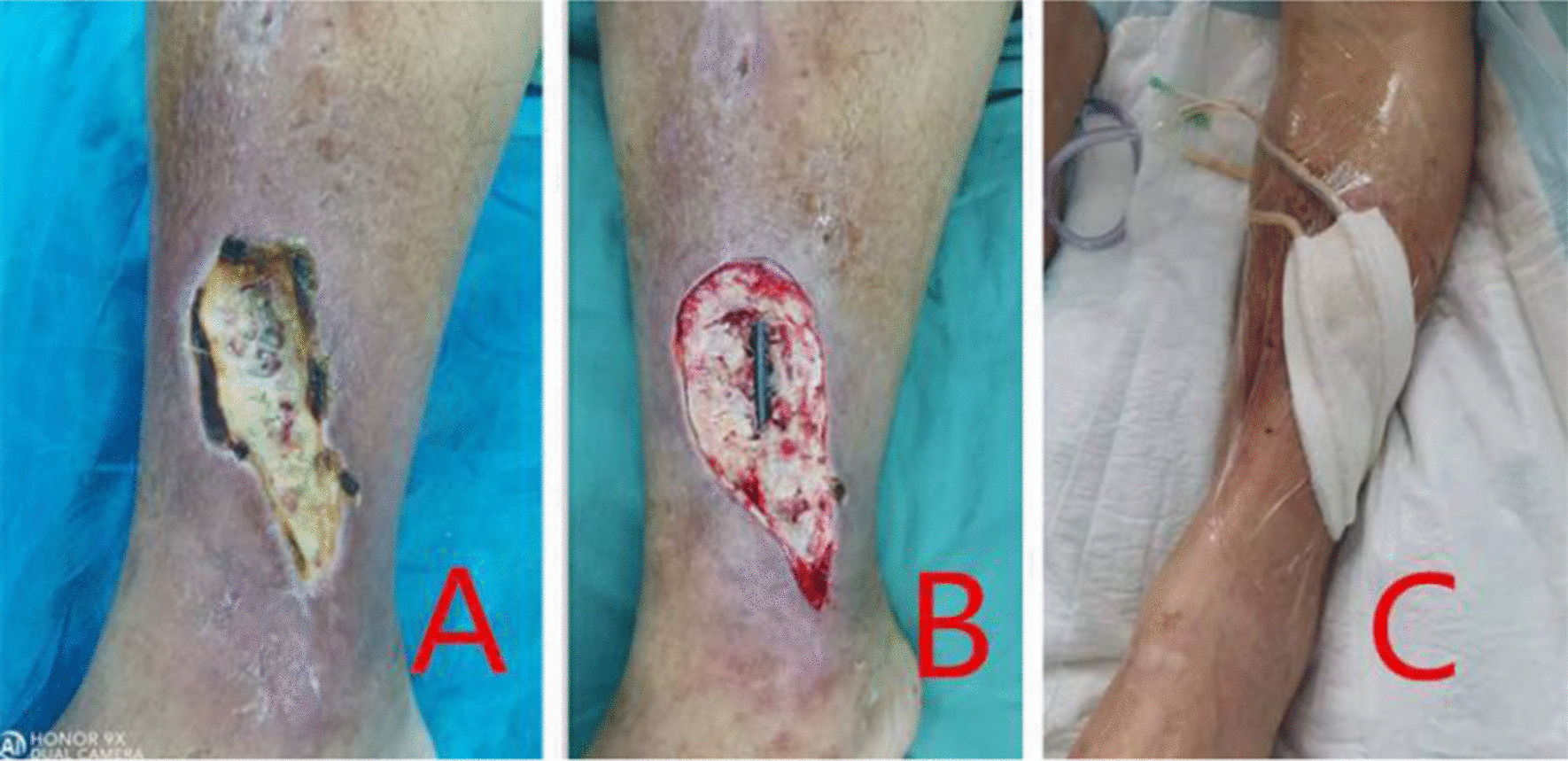
**A** Preoperative, **B** After the first stage of debridement, **C** VSD

After admission, the relevant examination was perfected, and the wound was subjected to bacterial culture and drug sensitivity test. In the first stage, the wound expansion was performed under general anesthesia or combined spinal–epidural anesthesia, removing the aging granulation tissue and fibrotic scared skin around the wound, removing the scared skin from the periphery of the steel plate or the anterior wall of the sinus to healthy soft tissue, the wound was fully opened and the sinus or subcutaneous cavities were eliminated. Removing the tendinous tissue such as necrotic tendons and ligaments around the joint of the wound base, and scrape off the granulation of the wound base until there is active bleeding. Careful removing the peptone in the steel plate hole and the steps and gaps formed between the edge of the steel plate and the bone surface, and then removing the peptone in the gap between the broken end of the fracture. After cleaning the wound again, the wound was externally applied with gauze impregnated with iodine voltfor 15 min, observing that there was no obvious bleeding, the wound was treated with VSD immediately or 24 h later. After 1–2 clinical periods of VSD till to obvious granulation development in the base of the wound, the second stage operation was performed (Fig. 1A–C).

#### The exposed joints and plates were covered with artificial dermis and grafted with fascial sleeve flap in stage II

**Fig. 2:**
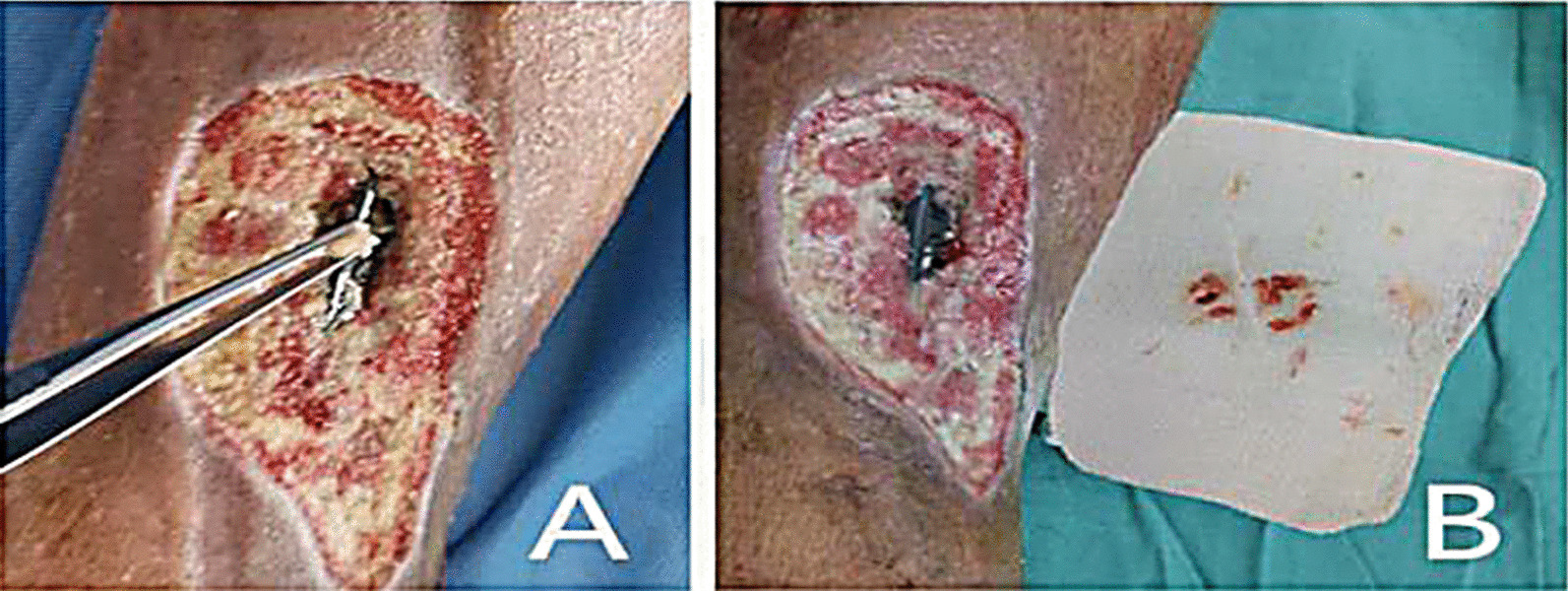
**A**, **B** Peptone in the gap of steel plate

After successful anesthesia, clean the wound again, the denatured and necrotic bone cortex was chiseled off 1 mm to 2 mm, the peptone in the step and gap formed between the edge of the steel plate and the bone surface and the peptone in the gap between the broken end of the fracture were removed (Fig. 2A, B).

**Fig. 3:**
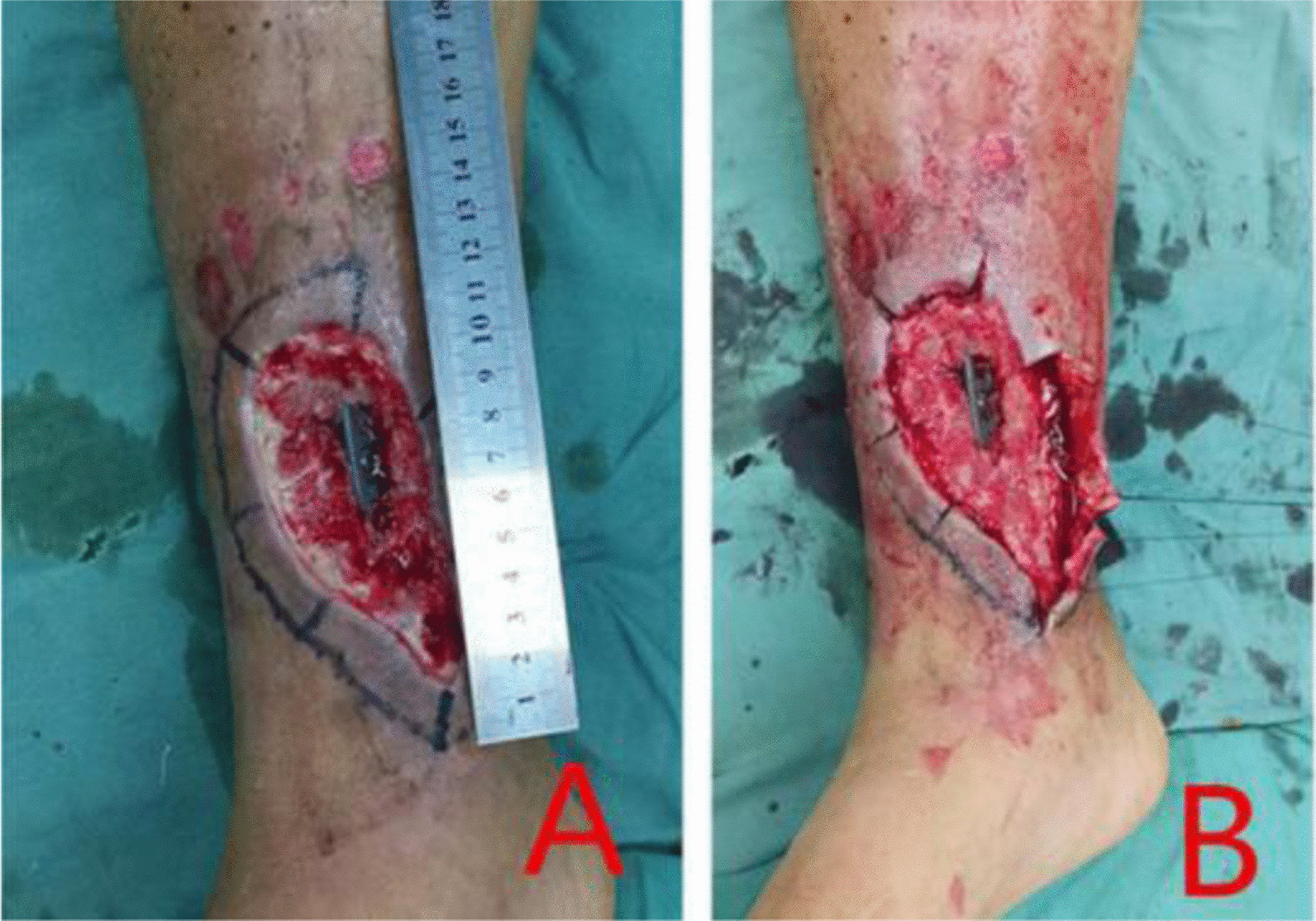
**A** Sleeve design, **B** Lift the sleeve

**Fig. 4:**
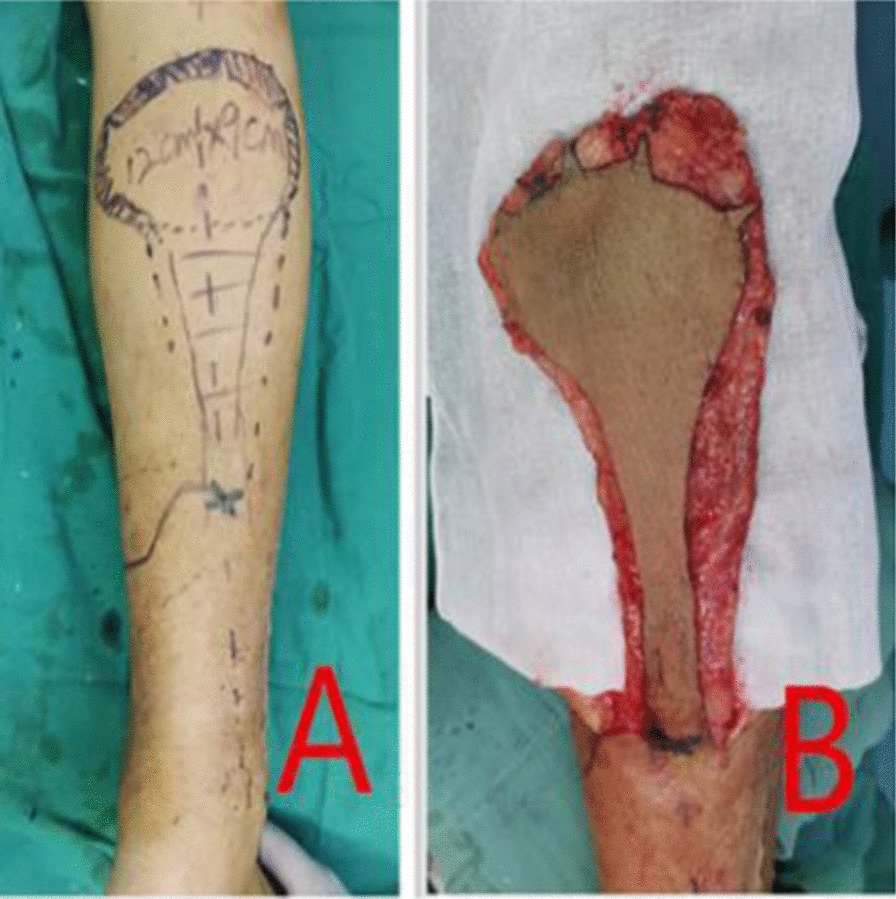
**A** Design of fascial sleeve flap, **B** Fascial sleeve flap

**Fig. 5:**
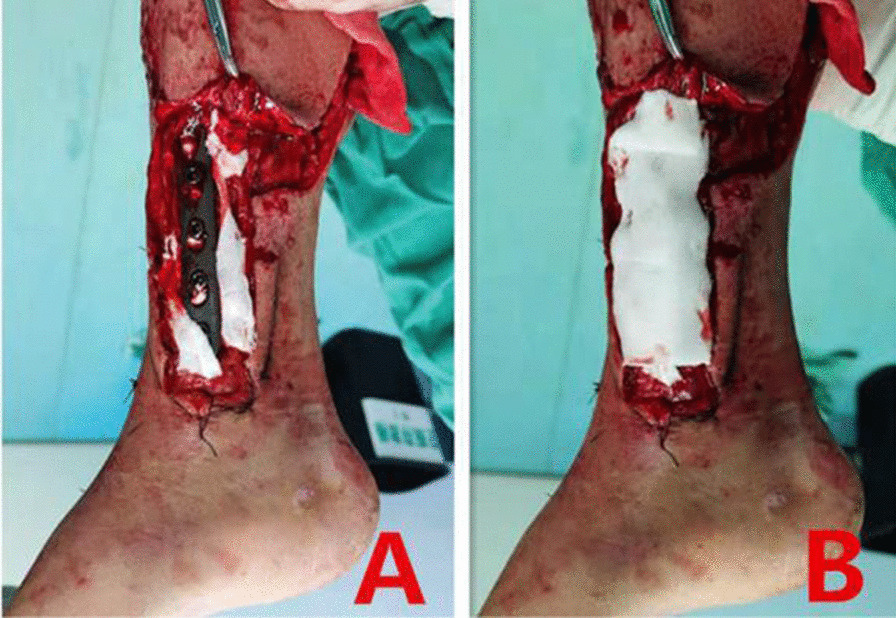
**A** Filling the pores and gaps of steel plate with artificial dermis, **B** Cover the exposed steel plate with artificial leather

The skin around the wound was lifted about 1 cm from the superficial layer of the deep fascia to the periphery to form a cuff. If the skin around the wound is scarred or severely adhered to the base, the epidermis of the wound margin can also be cut off about 1 cm width to form a fresh base (Fig. 3A, B), and after the exact hemostasis, the wound is washed with 75% alcohol, and the skin flap is designed according to the size of the wound. The flap was 1 cm enlarged than the designed area and in this expanded range, it is peeled off outward along the subdermis to form a fascia surface, cut along the outer edge of the fascia surface to the deep fascia layer, and lift the flap. A fascial sleeve of about 1 cm except the pedicle was formed in the periphery of the flap (Fig. 4A, B). If the cuff is not formed during debridement, there is no need to form a fascia sleeve. After the flap was lifted to cover the wound completely, the exposed bone and steel plate were covered with artificial dermis. The holes in the steel plate and the steps formed at the edge of the steel plate and the gaps between the steel plate and the bone should be filled closely in order to eliminate the dead cavity after transplantation (Fig. 5A, B). During suture, a vertical incision was made every 5 cm at the skin edge of the vertical cuff in order to reduce tension and facilitate drainage, and the flap was transferred to the recipient area, then the fascial sleeve was buried in the cuff around the wound. One or two rubber patches were placed under the flap, then, interrupted suture was used in the dermal margin of the flap and the wound to close the wound. Fllowing, the flap was bandaged under proper pressure. After the operation, lie in bed strictly for 2 weeks, raise the operative limbs to prevent pressure on the flap and pedicle, and routinely perform blood volume replenishment, use anti-inflammatory, relieve pain, keep warm, etc. The dressings were changed for the first time on the 3rd day after operation, and the survival of the flap was observed. Sural neurovascular flap was performed in six cases and lateral supramalleolar artery perforator flap in one case.

## Results

All the flaps survived well in 11 cases, without marginal necrosis and poor blood supply of the flap. During a follow-up for 6 months or till to the steel plate was removed, there was no bone plate exposure or sinus formation.

## Typical cases

**Fig. 6:**
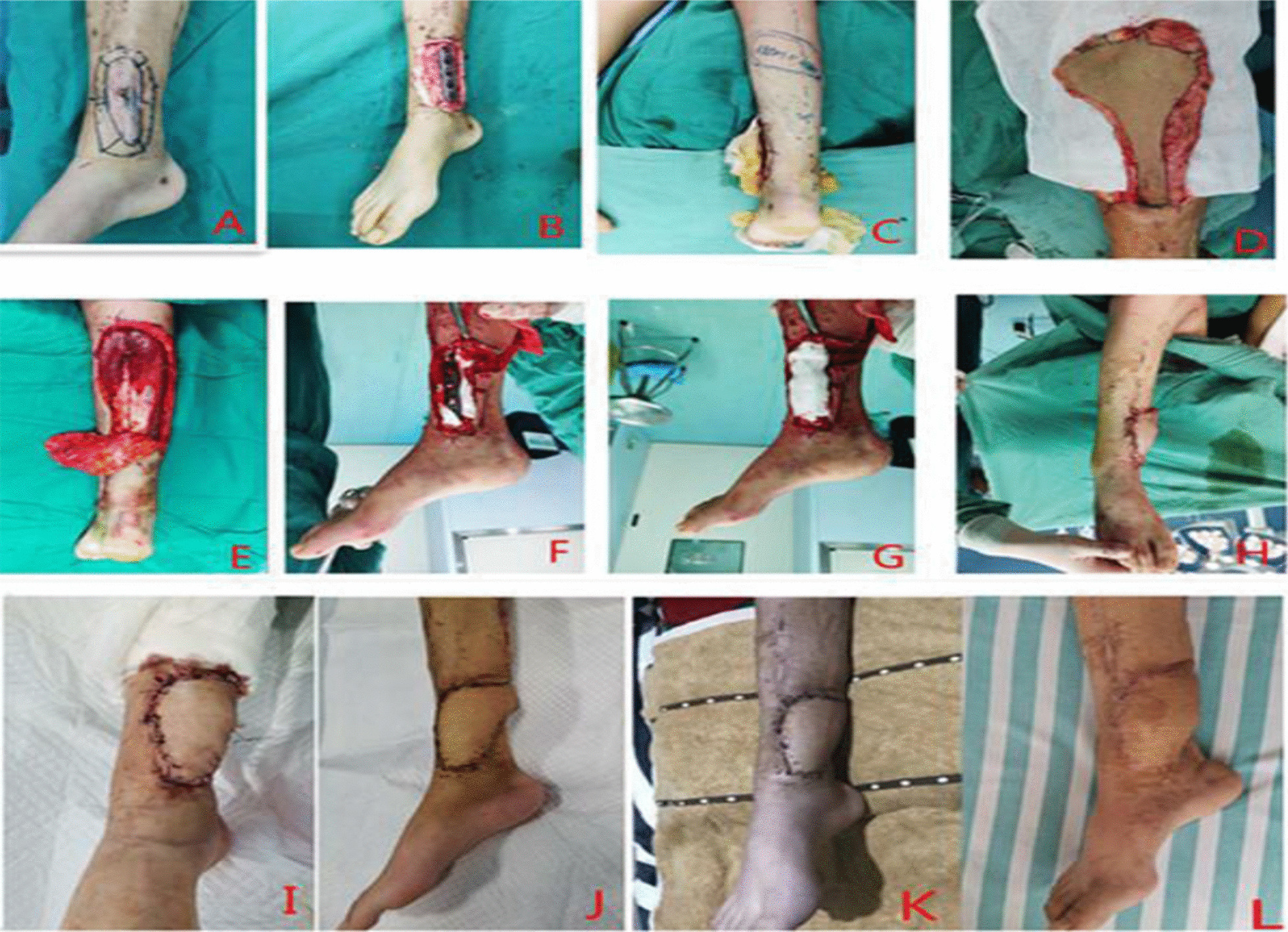
**A** Preoperative, debridement and incision design, **B** Expand the wound, lift the sleeve skin, and clean the peptone in the gap between the steel plates, **C** Design of sural nerve fascial sleeve flap, **D** Fascial sleeve flap, **E** Flap lifting, **F** Filling steel plate pores with artificial dermis, **G** Artificial dermis embedded steel plate, **H** Immediate treatment of flap transfer, **I** 3 days after operation, **J** 2 weeks after operation, **K** 1 month after operation, **L** 4 months after operation

A female patient with right tibiofibular fracture was treated with internal fixation with steel plate. After the operation, the skin was necrotic and ruptured, resulting in the exposure of steel plate. After debridement, artificial dermis covered exposed steel plate, sural neurovascular fascia sleeve flap transplantation, the flap survived well, followed up for 6 months, there was no rupture and sinus formation (Fig. 6A–L).

## Discussion

Orthopedic or emergency surgery is often used in the early stage of lower limb trauma [6–9], some patients have primary injuries, such as electric burn, severe rolling of skin and soft tissue, vascular injury or local hematoma, fat liquefaction, hematoma infection, etc., which eventually develop into skin necrosis [10–12].

For fracture patients, plate internal fixation is a more common method. However, the bone or steel plate was exposed due to postoperative skin swelling, excessive shear stress, skin necrosis or secondary wound infection, osteomyelitis and other reasons. Some scholars reported that if the wound fails to heal in 4 weeks, it will become a chronic wound. The important characteristic of chronic wound is the stagnation of wound healing process. Once a chronic wound is formed, the tissue repair process stops, the tissue around the wound becomes fibrotic, scarred, and stops growing. The exposed bone, tendon or steel plate can not be effectively covered by tissue, resulting in lasting exposure. For this kind of wound, the traditional repair method is to use various skin flaps. However, in some patients, the steel plate at the suture between the flap edge and the receiving area cracked again, and the steel plate was exposed again. In other patients, the flap healed well in the early stage, but 1 or 2 months after operation, the sinus appeared again on the suture between the flap and the recipient area, the secretion was discharged intermittently, and the sinus orifice showed typical chronic inflammation [13]. The swelling of the flap is an important reason for the re division of the flap and the exposure of bone steel nails in the later stage. Due to the contracture of the flap tissue and the tissue around the wound, the tension between the flap and the wound suture gradually increases, because the suture between the flap and the recipient tissue is a single end-to-end contact, due to the effect of tension, the tissue at the suture becomes thinner and finally splits, in particular, when the suture part crosses the bone or steel plate, there is not enough tissue in the deep layer to provide healing, and the wound will eventually crack again.

**Fig. 7:**
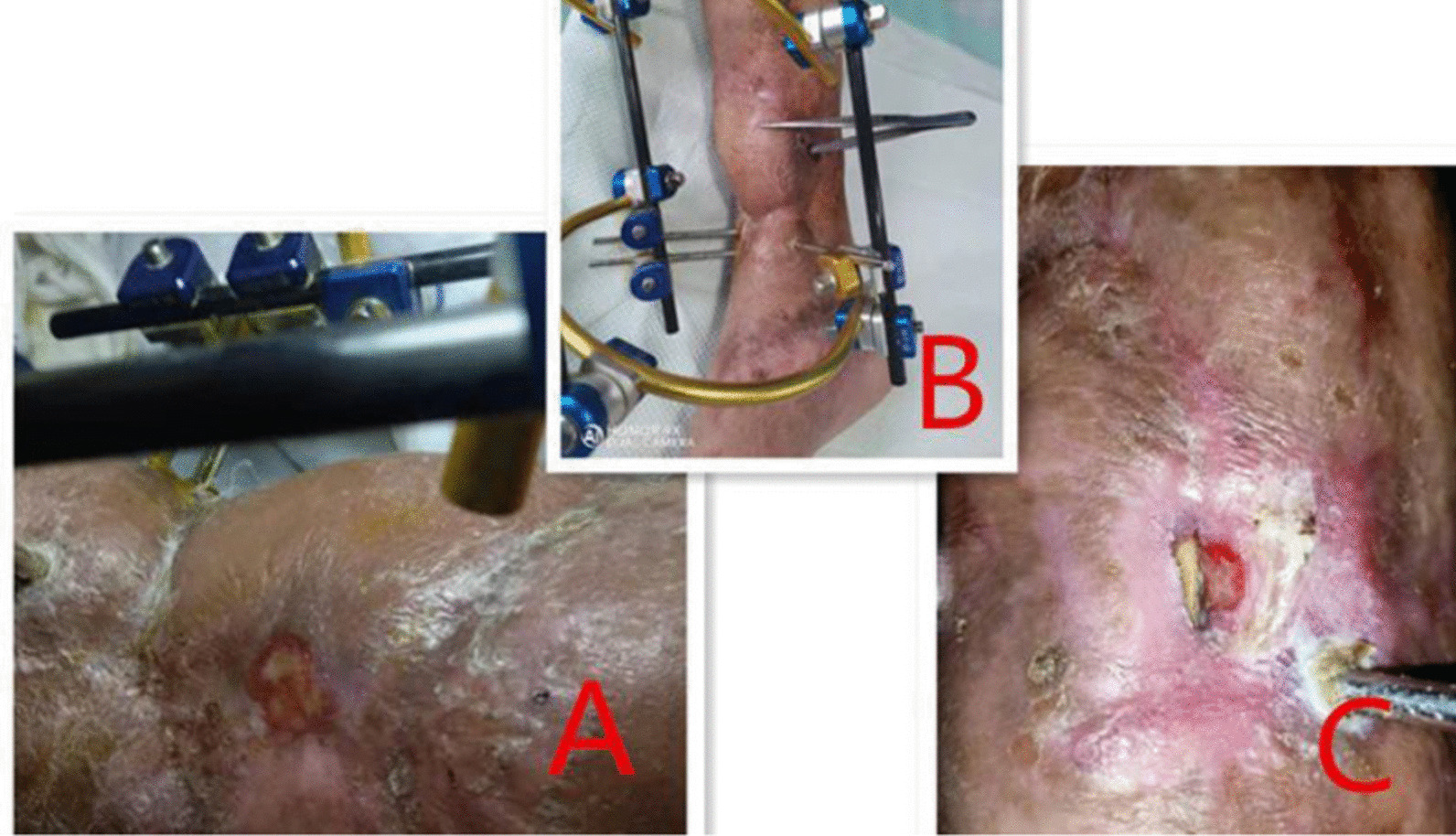
**A**–**C** The sinus was formed again after flap covering

In addition, due to the retention of foreign bodies such as dead bone, necrotic and degenerative tendon tissue and steel plate, the ejection reaction of the body also produces inflammatory exudation, which contains the colloidal substances such as proteins in the exudate precipitate and solidify in the foreign body surface and the surrounding spaces to form peptone which becomes a good culture medium for bacteria. Bacteria multiply in large numbers in the above-mentioned areas (Fig. 2), release toxins, and further aggravate the inflammatory reaction of the tissue around the wound, so that the wound continues to produce exudate and pus, forming a vicious circle, and the wound can not heal [14, 15]. After timely wound dressing change and anti-infective treatment, the acute inflammation of the wound was control. Due to the stimulation of chronic inflammation, the tissue around the wound gradually fibrosis, scarring, congestion and blood stasis, thickening, dark purple color, lack of elasticity, poor circulation, which is not conducive to wound repair [16–21] led and turned to chronic wound, the bone plate was exposed or formed one or more sinuses (Fig. 7A–C). After several months or even longer treatment, there was no obviously tending to heal.

In this study, according to the causes of secondary wound with chronic bone exposure, through one-stage expansion, remove the scar skin within a certain range around the wound, remove or remove the sinus wall, scrape the basal granulation tissue, remove the fresh wound bed in combination with VSD culture, change the chronic wound into acute wound, restart the wound healing process, and provide a good foundation for flap healing and wound final healing. A certain width of recipient granulation wound is formed around the wound, which can increase the contact surface between the transplanted flap and the base, so that the blood supply between the flap and the wound can be established quickly, it is conducive to the survival of flap and promote wound healing.

After the wound bed is ready, a second stage debridement is performed. The necrotic bone, periosteum, tendon tissue and peptone on the surface of the plate and bone were removed, and the space around the plate was filled with artificial dermis to completely cover the exposed bone and plate, then the wound was closed with flap. Artificial dermis isolated the contact between body tissue and foreign bodies at an early stage and blocked rejection [22]. The flap and periwound blood vessels can quickly grow into the artificial dermis and close the dead space around the bone plate proper pressure wrapping of the flap after operation can effectively prevent the possibility of residual dead space and purulent cavity formed by blood accumulation under the flap. The suture between the flap edge and the wound edge tissue was changed from end-to-end plane contact to three-dimensional mortise and tenon contact, this three-dimensional contact increases the contact area between the flap and the tissue around the wound, so that the flap and wound can establish blood supply in an earlier and wider range, the three-dimensional mortise and tenon contact can effectively reduce the risk of skin thinning and cracking at the suture caused by excessive tension caused by flap retraction. Since the bacteria in chronic wounds are mainly low toxic strains [23], the bacterial content is greatly reduced after twice debridement and cleaning with VSD and alcohol. Fresh wound granulation tissue and flap tissue have reliable blood supply, can completely remove residual bacteria, and significantly reduce the possibility of infection and sinus formation.

When expanding the wound, the scar skin and fibrotic tissue of the outer wall of the steel plate should be excised. If there is a sinus, the outer wall of the sinus should be incision, and the fibrous capsule inside the outer wall and the sinus cavity should be removed [9]. If necessary, make a transverse incision at the bottom of the sinus wall, so that the sinus wall can be loosely attached to the base after the fibrous capsule is removed. If the fibrotic scar of the outer wall of the sinus is so severe that it can not be well attached to the base, it can be removed as appropriate. Free bone fragments should be removed. The necrotic periosteum and tendinous tissue at the base of the wound were also removed as clean as possible. Scratching the aged granulation at the bottom can cause bleeding in the wound, which can lead to fresh wounds, thus activating the wound healing process. The protein-like tissue attached and precipitated in the holes of the steel plate, in the steps formed between the edge of the steel plate and the bone surface, in the gap formed by the incompact adhesion between the steel plate and the bone, and in the gap at the broken end of the bone should be carefully cleaned to remove. During the skin flap transplantation, the denatured and necrotic bone cortex should be chiseled off 1–2 mm, the peptone in the steel plate steps and spaces should be removed, and the exposed bone or steel plate should be covered with artificial dermis. The gap between the steps formed at the edge of the steel plate and the bones under the plate should be closely filled, so that the dead space will not remain after the flap transplantation and avoid infection and necrosis caused by effusion under the flap after operation.

Artificial dermis was developed by Yannas and Burke in 1980. It is a membrane artificial skin and a synthetic tissue used to repair skin tissue [24, 25]. Suzuki [26] researched and improved the product and developed a new artificial dermis (PELNAC), which is widely used in scar shrinkage repair, excision of wounds caused by tumors, traumatic skin defects, peeling of skin flaps and other reasons, the effect evaluation shows that the good rate is 86%. In recent years, PELNAC has been widely used in some wounds with bone exposure after trauma. When the artificial dermis was transplanted to the wound with poor basal blood supply, the time of local vascularization was delayed than that of the wound with good basal blood supply [27]. In this study, two debridement and VSD were performed to complete the wound bed preparation, so as to form a wide base with good blood supply around the wound, so as to ensure the vascularization of artificial dermis and the healing of flap. The blood supply of the flap is reliable. When the flap covers the artificial dermis, in a short time, the deep layer of the artificial dermis is filled with new collagen, and the sponge structure is replaced by true skin like tissue, so as to quickly complete the embedding process of steel plate or exposed bone.

## Data Availability

We declare that the manuscript, including relevant data, figures and tables, is true and reliable. All data generated or analysed during this study are included in this published article [and its additional information files].
